# KNIME-CDK: Workflow-driven cheminformatics

**DOI:** 10.1186/1471-2105-14-257

**Published:** 2013-08-22

**Authors:** Stephan Beisken, Thorsten Meinl, Bernd Wiswedel, Luis F de Figueiredo, Michael Berthold, Christoph Steinbeck

**Affiliations:** 1 European Molecular Biology Laboratory - European Bioinformatics Institute (EMBL-EBI), Wellcome Trust Genome Campus, Hinxton, Cambridge, UK; 2 Nycomed Chair for Bioinformatics and Information Mining, University of Konstanz, Konstanz, Germany; 3 KNIME.com AG, Technoparkstr. 1, 8005 Zürich, Switzerland

**Keywords:** Cheminformatics, Workflows, Data integration, Software library

## Abstract

**Background:**

Cheminformaticians have to routinely process and analyse libraries of small molecules. Among other things, that includes the standardization of molecules, calculation of various descriptors, visualisation of molecular structures, and downstream analysis. For this purpose, scientific workflow platforms such as the Konstanz Information Miner can be used if provided with the right plug-in. A workflow-based cheminformatics tool provides the advantage of ease-of-use and interoperability between complementary cheminformatics packages within the same framework, hence facilitating the analysis process.

**Results:**

KNIME-CDK comprises functions for molecule conversion to/from common formats, generation of signatures, fingerprints, and molecular properties. It is based on the Chemistry Development Toolkit and uses the Chemical Markup Language for persistence. A comparison with the cheminformatics plug-in RDKit shows that KNIME-CDK supports a similar range of chemical classes and adds new functionality to the framework. We describe the design and integration of the plug-in, and demonstrate the usage of the nodes on ChEBI, a library of small molecules of biological interest.

**Conclusions:**

KNIME-CDK is an open-source plug-in for the Konstanz Information Miner, a free workflow platform. KNIME-CDK is build on top of the open-source Chemistry Development Toolkit and allows for efficient cross-vendor structural cheminformatics. Its ease-of-use and modularity enables researchers to automate routine tasks and data analysis, bringing complimentary cheminformatics functionality to the workflow environment.

## Background

The routine work of a cheminformatician involves the processing of libraries of small molecules. Standardising molecules, e.g., adding hydrogens or removing unconnected structures, calculation of molecular descriptors, and visualisation of chemical structures in two- or three-dimensional space are just a few examples of recurrent tasks that are carried out upstream of cheminformatic pipelines. Several free cheminformatics libraries and tools have been developed to deal with these tasks, such as the CDK [1], RDKit [2], and OpenBabel [3] to mention only a few.

Typically, building a comprehensive pipeline for small molecules requires a basic understanding of a scripting language to concatenate input and output from different tools or call functions from a cheminformatics library. For experimental scientists, usage of APIs (application programming interfaces) or programming languages adds a constraint to more in-depth analysis. On the other hand, standalone tools suffer from their limited scope. Even simple tasks like the visual characterisation of a chemical library [4] requires importing and exporting data in various formats using different tools.

Workflow environments circumvent the above mentioned challenges to various degrees by providing a common platform for different tools and have become increasingly popular with the scientific community [5,6]. The Konstanz Information Miner (KNIME) [7] is an open-source workflow platform that supports a wide range of functionality and has an active cheminformatics/bioinformatics community, e.g., with plug-ins for next generation sequencing or image analysis [8-10]. For a detailed description of the KNIME data analysis platform see [11].

The cheminformatics plug-in KNIME-CDK is based on the Chemistry Development Kit (CDK), an open-source cheminformatics library. It wraps elements of the library’s core functionality and exposes it to the user. In contrast to other cheminformatics plug-ins available in KNIME, the project and its core library are fully open and community-driven.

## Implementation

KNIME-CDK has been developed in Java 1.6 and is available via the KNIME update mechanism. The plug-in including its sources is available as release (stable) build and nightly (pre-release) build under GNU LGPL v3. It has been tested on KNIME Desktop version 2.6 and 2.7, the latter uses Java 1.7, with 2 GB memory and default settings otherwise, using the ChEBI compound library [12]. Over the last year, the plug-in and its underlying core library have been updated, reducing memory requirements and improving overall performance. The KNIME-CDK community site and forum [13] provide an overview of the implemented functionality and support respectively.

Following KNIME’s data model, the individual CDK molecule representations are stored in their own data cell type, the atomic unit for tabular data transfer from one node to another. A node can be considered as single worker carrying out a single function. Here node names are written in italic. Data persistence is guaranteed via the Chemical Markup Language (CML) [14] serializing the molecule when necessary. The underlying CDK molecules are handled and stored within data cells in standardized form, i.e., with implicit hydrogen atoms added, atom types perceived, and aromaticity detected. This guarantees consistency across all nodes and simplifies usability of the plug-in by hiding technical details from the user, hence allowing the scientist to focus on the task at hand.

The plug-in accepts molecules in CML, SDFile, MDL Mol, InChI, and SMILES formats [15] via the *Molecule to CDK* node and writes SDFiles via the *CDK to Molecule* node, hence converting the CDK molecule back to the default SDFile cell, that can be used with other cheminformatics plug-ins. In addition, the implemented SDFile interface ensures that all SDFile cell accepting nodes can directly be connected to KNIME-CDK nodes.

**Figure 1:**
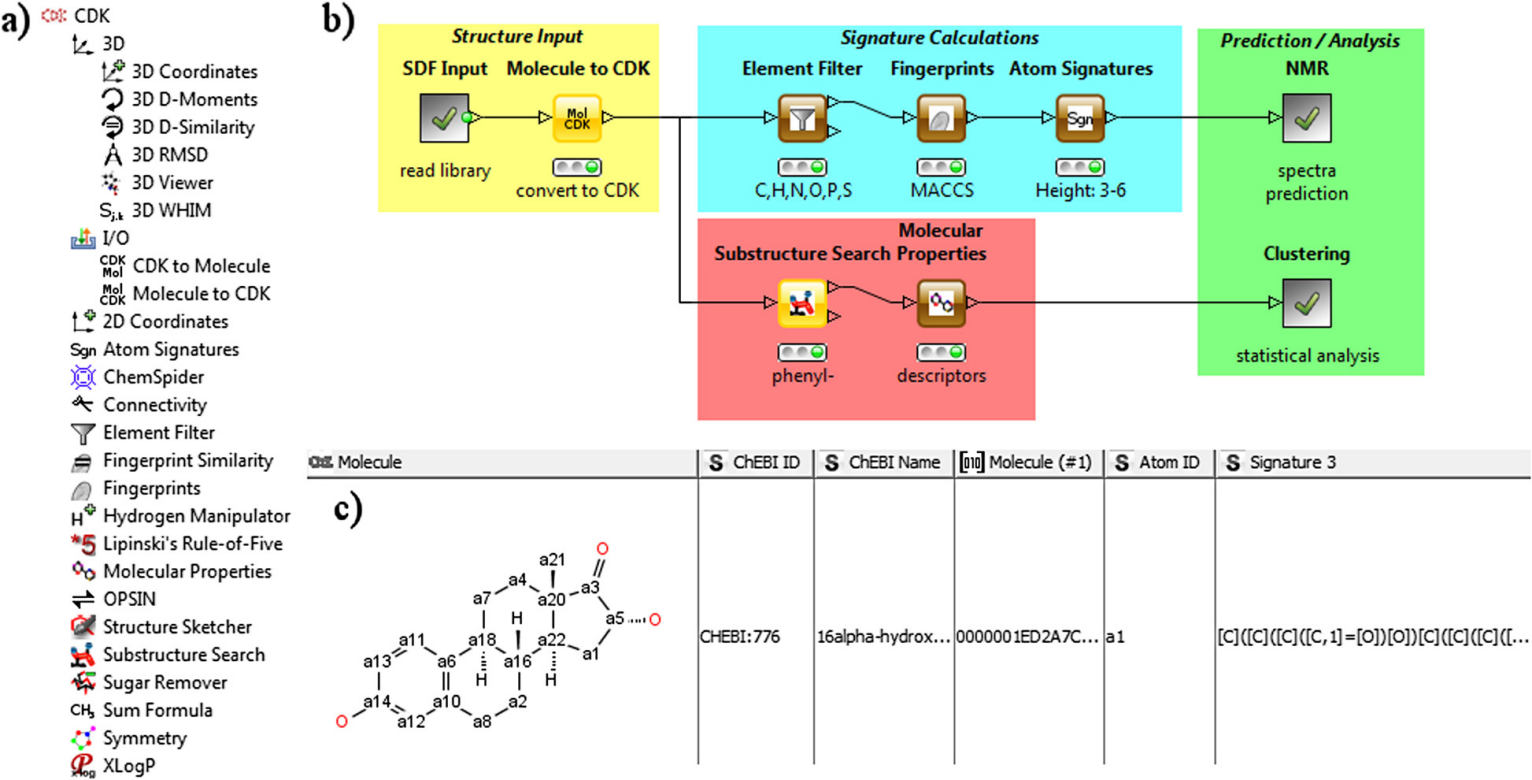
**Example workflow and out-port views.** Overview of the KNIME-CDK plug-in. **a)** View of the node repository showing all available nodes. **b)** Example workflow for descriptor calculation. The molecule library is read in and filtered for structures containing phenol groups before counting the number of hydrogen donors and acceptors (lower path). Simultaneously, MACCS fingerprints and atom signatures are calculated for the atom-filtered molecules (upper path). **c)** Example row from the out-port view of the *Atom Signatures* node.

All subsequent operations are carried out on the internal CDK molecule representation and include, *inter alia*, generation of coordinates, atom signatures of various heights, common fingerprints, e.g., MACCS and Pubchem, two- and three-dimensional molecular descriptor values including XLogP and Lipinski’s Rule of Five, chemical name lookup via OPSIN [16], and substructure search (Figure 1a). Different routes in a workflow can run in parallel and nodes run always multi-threaded. In Figure 1b a chemical library is filtered for molecules containing a phenol group before successive hydrogen acceptor / donor count while being used for MACCS fingerprint and atom signature generation. The out-port view, i.e., the resulting data table, is shown for the *Atom Signatures* node. Further use cases of workflows using the KNIME-CDK plug-in include the management and analysis of chemical libraries through molecular descriptors, conformer analysis via RMSD, and NMR spectra prediction. Example workflows for these tasks can be found in the repository [17] of the myExperiment virtual research environment [18].

Complementing the signature node, the KNIME preference page contains a CDK tab to set global visualisation preferences. Given two- or three-dimensional coordinates, a renderer is provided to draw the molecules using the JChemPaint library [19]. By default the element symbol is drawn. The preference page allows to draw either canonical or sequential atom numbers instead of either all atoms or carbon / hydrogen atoms only.

## Discussion

The KNIME-CDK plug-in was tested using the structurally diverse ChEBI library with a total of 23,240 3-star structures. For testing purposes, the library was used in SDFile format, release 98, because this could arguably be considered the most common use case. For comparison, the well-established RDKit plug-in was used. Using the ChEBI SDFile, consistent input-serialization-output was tested using round tripping to ensure that no information is lost or altered.

From the 23,240 structures, 22,225 structures (95.6%) could successfully be read in, marginally less than with the RDKit plug-in (22,482 structures, 96.7%). Not all molecules could be converted into the CDK representation because some classes are not supported throughout the node’s read process. Currently the following groups lack support (examples in brackets): Coordination entities [CHEBI:16304], ’exotic’ atoms [CHEBI:27698], complexed porphyrins [CHEBI:27888], some radical species [CHEBI:33101, CHEBI:33105], and repeated structures [CHEBI:65304]. The structures were read in 43.0±4.5 seconds compared to 12.0±0.7 seconds (RDKit). Even though the KNIME-CDK plug-in is not as fast as RDKit, which uses a native C++ implementation, its functionality should be seen as complimentary to other plug-ins available and its speed is still adequate.

The ChemAxon Marvin Extensions Feature, 2.6.3.v0135, was used to create canonical SMILES from the structures that were loaded with KNIME-CDK and RDKit. For 2794 (12.6%) structures different SMILES were produced, due to the fact that different internal representations and the nature of the problem, inescapably produces variation. This highlights one of the benefits of employing more than one library for processing and analysis tasks. In addition, KNIME-CDK offers some unique functionality including various molecular descriptors, fingerprints, and equivalent class calculation.

With that knowledge, KNIME-CDK can be used for chemical data exploration in synergy with other cheminformatics plug-ins to make proper use of the framework environment. We will continue to update the plug-in continuously to take the newest developments of the CDK project into account. New nodes will be added on a “on-demand” basis.

## Conclusions

We presented KNIME-CDK, an open-source cheminformatics plug-in for the KNIME platform. The plug-in brings additional cheminformatics functionality to the platform based on a community-driven open library. Functionality includes molecule conversion to and from common formats, substructure searching, generation of signatures, fingerprints, and molecular properties. It supports the typical range of organic chemical structures similar to RDKit but adds new functionality to the framework. The plug-in is easy to use and enables the community to build further nodes based on the popular CDK library that work in combination with the existing molecule representation. Issues that will be addressed are the overall speed and input capability of the plug-in to make it more usable and better suited for high-throughput analysis.

## Availability and requirements

•**Project name:** KNIME-CDK

•**Project URL:**http://tech.knime.org/community/cdk

•**Availability:** All sources and compiled code are available via the KNIME update mechanism.

•**Operating system(s):** Platform independent

•**Programming language:** Java 1.6

•**Other requirements:** KNIME Desktop v2.6+

•**License:** GNU LGPLv3

## Competing interests

The authors declare that they have no competing interests.

## Authors’ contributions

TM and BW wrote the initial plug-in. SB and LF updated the project and are the current administrators. They deal with all community requests and ensure full functionality of the plug-in. SB wrote the manuscript. CS designed the core library and provides continuous support. All authors read and approved the final manuscript.

## References

[B1] Steinbeck C Han Y Kuhn S Horlacher O Luttmann E Willighagen E The Chemistry Development Kit (CDK): an open-source Java library for Chemo- and Bioinformatics J Chem Inf Comput Sci 2003 43 2 493 500 [http://www.ncbi.nlm.nih.gov/pubmed/16796559] 10.1021/ci025584y 12653513 PMC4901983

[B2] Landrum G RDKit: Open-source cheminformatics [http://www.rdkit.org/]

[B3] O’Boyle NM Banck M James Ca Morley C Vandermeersch T Hutchison GR Open Babel: An open chemical toolbox J Cheminformatics 2011 3 33 [http://www.ncbi.nlm.nih.gov/pubmed/21982300] 10.1186/1758-2946-3-33 PMC3198950 21982300

[B4] Le Guilloux V Colliandre L Bourg S Guenegou G Dubois-Chevalier J Morin-Allory L Visual characterization and diversity quantification of chemical libraries. 1) Creation of delimited reference chemical subspaces J Chem Inf Model 2011 51 8 1762 74 [http://www.ncbi.nlm.nih.gov/pubmed/21761916] 10.1021/ci200051r 21761916

[B5] Magalhaes WCS Machado M Tarazona-santos E A graph-based approach for designing extensible pipelines BMC Bioinf 2012 13 163 163 10.1186/1471-2105-13-163 PMC3496580 22788675

[B6] Warr Wa Scientific workflow systems: pipeline pilot and KNIME J Comput-aided Mol Des 2012 26 7 801 4 [http://www.ncbi.nlm.nih.gov/pubmed/22644661] 10.1007/s10822-012-9577-7 22644661 PMC3414708

[B7] Berthold MR Cebron N Dill F Gabriel TR Kötter T Meinl T Ohl P Sieb C Thiel K Wiswedel B KNIME: The Konstanz Information Miner Studies in Classification, Data Analysis, and Knowledge Organization (GfKL 2007) 2007 Heidelberg-Berlin: Springer-Verlag

[B8] Jagla B Wiswedel B Coppée JY Extending KNIME for next-generation sequencing data analysis Bioinf (Oxford, England) 2011 27 20 2907 9 [http://www.ncbi.nlm.nih.gov/pubmed/21873641] 10.1093/bioinformatics/btr478 21873641

[B9] Lindenbaumm P Le Scouarnec S Portero V Redon R Knime4Bio: a set of custom nodes for the interpretation of next-generation sequencing data with KNIME Bioinf (Oxford, England) 2011 27 22 3200 1 [http://www.pubmedcentral.nih.gov/articlerender.fcgi?artid=3208396] 10.1093/bioinformatics/btr554 PMC3208396 21984761

[B10] Strobelt H Bertini E Braun J Deussen O Groth U Mayer TU Merhof D HiTSEE KNIME: a visualization tool for hit selection and analysis in high-throughput screening experiments for the KNIME platform BMC Bioinf 2012 13 Suppl 8 Suppl 8 S4 [http://www.pubmedcentral.nih.gov/articlerender.fcgi?artid=3355333] 10.1186/1471-2105-13-S8-S4 PMC3355333 22607449

[B11] KNIME KNIME - Professional open-source software [http://www.knime.com/]

[B12] Hastings J de sMatos P Dekker a Ennis M Harsha B Kale N Muthukrishnan V Owen G Turner S Williams M Steinbeck C The ChEBI reference database and ontology for biologically relevant chemistry: enhancements for 2013 Nucleic Acids Res 2012 41 November 2012 456 463 [http://www.nar.oxfordjournals.org/cgi/doi/10.1093/nar/gks1146] 10.1093/nar/gks1146 PMC3531142 23180789

[B13] KNIME KNIME Community site [http://tech.knime.org/community/cdk]

[B14] Kuhn S Helmus T Lancashire RJ Murray-Rust P Rzepa HS Steinbeck C Willighagen EL Chemical markup, XML, and the world wide web. 7. CMLSpect, an XML vocabulary for spectral data J Chem Inf Model 2007 47 6 2015 34 [http://www.ncbi.nlm.nih.gov/pubmed/17887743] 10.1021/ci600531a 17887743

[B15] Warr W Representation of chemical structures Wiley Interdisciplinary Rev: Comput 2011 1 August 557 579 [http://onlinelibrary.wiley.com/doi/10.1002/wcms.36/full]

[B16] Lowe DM Corbett PT Murray-Rust P Glen RC Chemical name to structure: OPSIN, an open source solution J Chem Inf Model 2011 51 3 739 53 [http://www.ncbi.nlm.nih.gov/pubmed/21384929] 10.1021/ci100384d 21384929

[B17] MyExperiment MyExperiment KNIME workflow [http://www.myexperiment.org/workflows/3045.html]

[B18] Goble Ca Bhagat J Aleksejevs S Cruickshank D Michaelides D Newman D Borkum M Bechhofer S Roos M Li P De Roure D myExperiment: a repository and social network for the sharing of bioinformatics workflows Nucleic Acids Res 2010 38 Web Server issue W677 82 [http://www.pubmedcentral.nih.gov/articlerender.fcgi?artid=2896080] 20501605 10.1093/nar/gkq429 PMC2896080

[B19] Krause S Willighagen E Steinbeck C JChemPaint-using the collaborative forces of the internet to develop a free editor for 2D chemical structures Molecules 2000 5 1 93 98 [http://www.mdpi.com/1420-3049/5/1/93] 10.3390/50100093

